# No retinopathy after orbital irradiation in patients with thyroid eye disease – a follow up study

**DOI:** 10.3389/fendo.2025.1720650

**Published:** 2025-12-10

**Authors:** Sara Makhoul, Janos K. Aranyosi, Emese Csiki, Hilda Urbancsek, Edit B. Nagy, Annamaria Erdei, Mariann Fodor, Arpad Kovacs, Endre V. Nagy, Bernadett Ujhelyi

**Affiliations:** 1 Department of Ophthalmology, Faculty of Medicine, University of Debrecen, Debrecen, Hungary; 2 Doctoral School of Clinical Medicine, University of Debrecen, Debrecen, Hungary; 3 Department of Oncoradiology, Faculty of Medicine, University of Debrecen, Debrecen, Hungary; 4 Department of Oncology, Faculty of Medicine, University of Debrecen, Debrecen, Hungary; 5 Divison of Radiology and Imaging Science, Department of Medical Imaging, Faculty of Medicine, University of Debrecen, Debrecen, Hungary; 6 Division of Endocrinology, Department of Internal Medicine, Faculty of Medicine, University of Debrecen, Debrecen, Hungary

**Keywords:** thyroid eye disease, radiotherapy, exophthalmos, retinopathy, diabetes

## Abstract

**Introduction:**

Thyroid eye disease (TED) is an autoimmune disease of the orbit. In moderate to severe active disease, orbital radiotherapy (ORT) is a treatment option. Diabetes is considered a relative contraindication because of potential induction of retinopathy. The purpose of this study was to decide if ORT remains a risk towards retinopathy in the era of linear accelerators with 3D computer aided design. Follow up examinations were performed to detect radiogenic retinopathy.

**Methods:**

Of the 151 patients who received ORT for TED between 2004–2020 at the University of Debrecen, 97 patients were available for follow up. In each case, 3D-based LINAC radiotherapy with a total dose of 20 Gy in 2 Gy daily fractions was administered. Eighteen patients had type 2 and one patient had type 1 diabetes. Median duration of diabetes was 16 (IQR:11.75-17.0) years; most recent HbA1c before irradiation was 6.55 (IQR:6.22-6.95) %. Ophthalmological follow up included slit-lamp and fundus examination, visual acuity, exophthalmometry and clinical activity score (CAS) calculation.

**Results:**

Median time between TED start and ORT was 2 (IQR:1-8) years. At the time of ORT, median TSH was 0.84 (IQR:0-1.41) mU/L, fT4 18 (IQR:12.15-25.40) pmol/L and fT3 6.5 (IQR:4.4-11.0) pmol/L, CAS was 5 (IQR:4-6). No diabetic retinopathy was present before ORT. The median follow-up period after completion of ORT was 60 (IQR:38-100) months. No patient developed retinopathy.

**Discussion:**

The risk of radiation-induced retinopathy after orbital irradiation, including well controlled diabetic patients, appears to be negligible. In the absence of pre-existing diabetic retinopathy and with good glycemic control ORT might be safe if linear accelerator with 3D planning is used. However, ophthalmic surveillance is still recommended.

## 1 Introduction

Thyroid eye disease (TED) is a condition associated with autoimmune thyroid disease (1). Symptoms of TED are proptosis, eye irritation, watery or red eyes, double vision and orbital pain (2, 3). Overall prevalence of TED is about 40 % in individuals with Graves’ disease. Ninety percent of cases are associated with hyperthyroidism (4). During the active phase of TED, the autoimmune process leads to thickening of extraocular muscles and expansion of orbital connective tissue (5). Oedematic swelling of orbital components is a major contributor to increased orbital pressure (6). The most severe condition, dysthyroid optic neuropathy (DON) is seen in 3-7% of the cases (7). In the late, inactive stage fibrosis predominates (8).

Management of TED is a collaborative work in TED centers; endocrinologist, ophthalmologist, radiologist, nuclear medicine specialist and physician with specialization in radiotherapy are members of the team. TED treatment options are summarized in recent guidelines (9, 10). Regardless of severity, it is important to adjust thyroid hormone levels to ensure continuous euthyroidism, immediately quit smoking, and use artificial tears and eye gels as lubricants to prevent corneal ulceration (11).

Orbital radiotherapy (ORT) is an effective, well-established treatment for TED (12). ORT has been used in the management of TED for over seven decades (12). For moderate to severe active TED, first-line treatment consists of intravenous steroid administration alone or in combination with ORT (13). ORT, when administered concurrently with systemic corticosteroids, can enhance the anti-inflammatory response, reduce orbital tissue oedema, and improve ocular motility compared with corticosteroid monotherapy (14). This regimen is considered to have synergistic effects and may improve disease outcomes (15, 16). In accordance with the consensus statement by the American Thyroid Association and the European Thyroid Association the combined regimen is an option in active, moderate-to-severe cases, especially if a previous course of systemic corticosteroid had failed to result in a sustained therapeutic effect (9, 10).

Most commonly used cumulative dose is 20 Gy, which is delivered in 10 fractions over 2 weeks (17). Technologies for external beam radiotherapy are linear accelerator (LINAC) and cobalt-60-based radiation therapies (18). Cobalt-60 radiation sources can result in greater radiation scatter (19). They have been gradually replaced by LINAC, and are not used since the early 2000s (20). LINAC generally delivers more controllable, more precisely targeted radiation, which is further improved by 3D planning (3D-CRT) and conformal radiation therapy (IMRT) techniques (21). The risk of retinopathy depends on the total dose (risk increases above 45–50 Gy with conventional fractioning), the dose of one fraction (risk increases if one fraction is above 2Gy) and is influenced by existing comorbidities (22). Retrospective data suggest that ORT, either alone or in combination with corticosteroids, may help manage compressive optic neuropathy in TED, while also improving diplopia and soft tissue swelling (23). Possible short-term side effects of irradiation include eye redness, periorbital swelling, and in some cases temporary worsening of symptoms (17). Long-term putative side effects are radiogenic retinopathy, early cataract formation, and radiation induced tumors (24). Development of radiogenic retinopathy has been described in several reports from the pre-linear accelerator era, which were linked to outdated equipment, improper techniques, excessive radiation doses, or the presence of diabetes (25). Few cases of radiogenic retinopathy have been reported in the absence of diabetes after the usual 20 Gy dose (25). Retinopathy may start 6–12 months after ORT completion, and once it appears, is usually irreversible. Typical fundus pathologies include vascular occlusions, dilatation of blood vessels, presence of collaterals and microaneurysms of capillaries (26). Histopathological evaluation reveals parenchymal inflammation, leukocyte infiltration in capillaries, glial hypertrophy, necrotic and gliotic lesions, neuronal swelling and degeneration (27). In the late phase, regenerated blood vessels, nonperfusion areas and ischemic fields are present (22).

Cataract formation is a common side effect of cobalt-60 irradiation. Before the linear accelerator era, the lens received 10% of the radiation dose, making it more susceptible to cataract formation (25).

Cautious patient selection for ORT considering strict criteria is essential. Untreated severe hypertension and pre-existing diabetic retinopathy are contraindications (28). Diabetes mellitus without ocular complications is a relative contraindication to treatment; professional guidelines currently recommend caution in this regard (9, 10). Pre-existing diabetic microangiopathy combined with radiation exposure may make the retina more prone to development of retinopathy by increasing vascular damage, endothelial injury, capillary occlusion, and retinal ischemia. Further, chronic hyperglycemia impairs DNA repair, disrupts endothelial and pericyte function, and increases oxidative stress (29).

The aim of the present study was to retrospectively analyze the late complications of ORT in TED patients with or without diabetes mellitus, treated between 2004 and 2020.

## 2 Materials and methods

Between 2004 and 2020, seven hundred and sixty-six patients were diagnosed with TED at the University of Debrecen. We are a tertiary referral center responsible for the care of patients from four counties in Hungary. One hundred and fifty-two patients underwent ORT for moderate to severe TED. Data on this cohort have not been previously reported. None of them had diabetic retinopathy or uncontrolled hypertension, as these were considered contraindications of ORT. Patients who had a history of radiotherapy involving the orbital region before or after ORT were excluded from the analysis.

During treatment and follow up, the standard interdisciplinary approach has been used (9, 10). For the present study, patients’ medical history, ophthalmological examination results and laboratory parameters including TSH, FT4, FT3, TRAb, and HbA1c were retrospectively analyzed, with particular attention to the presence of diabetes mellitus. During follow up after ORT, patients regularly underwent detailed ophthalmic evaluation including visual acuity testing, detailed slit-lamp examination, dilated ophthalmoscopy, intraocular pressure measurement and Hertel exophthalmometry. Ophthalmoscopy was performed by one of two ophthalmologists (SM, JKA), and the fundus photo was evaluated by the other. Potential discordant readings for the presence or absence of retinopathy would have been decided by a third ophthalmologist (BU). Disease activity was determined by means of the 10 or 7 item Clinical Activity Score (CAS), as appropriate (30). Patients with diabetes mellitus either had a record of being diagnosed with diabetes according to the then valid criteria, or were diagnosed during pre-ORT screening. Diabetic retinopathy screening was performed by indirect ophthalmoscopy with pupillary dilatation. Color fundus photography was performed according to ETDRS (standard seven field) and evaluated by two ophthalmologists. If the two opinions on the presence or absence of diabetic retinopathy were discordant, the decision was made by a senior expert.

Treatment for the patients was always provided according to the most recent guidelines. Non-contrast computed tomography (CT) simulation was performed prior to irradiation, with the patient immobilized in a dedicated thermoplastic mask to ensure reproducible head positioning. Target delineation and organ-at-risk (OAR) identification was carried out on 3D-based planning images. The clinical target volume (CTV) included the bilateral retrobulbar regions, carefully excluding the posterior contour of the eyeball and adjacent bony structures. The planning target volume (PTV) was generated by adding a 5 mm isotropic margin around the CTV to account for setup uncertainties. Treatment planning was performed using a three-dimensional conformal radiotherapy (3D-CRT) technique with two opposing photon fields of 6 MV energy, delivered by a linear accelerator (LINAC). A total prescribed dose of 20 Gy was administered to the PTV in 10 daily fractions of 2 Gy, delivered on consecutive weekdays. Image-guided radiotherapy (IGRT) verification was performed prior to each fraction to ensure accurate patient positioning and target coverage. During planning, organs at risk (OARs) including the ocular bulbs, lenses, and brain tissue were contoured, and dose constraints were applied according to the ‘Quantitative Analyses of Normal Tissue Effects in the Clinic’ (QUANTEC) recommendations (31, 32). The lenses, being the most radiosensitive structures in this region, were given the highest priority for dose sparing. A maximum lens dose of 7 Gy was applied at our institution as a planning constraint for all patients, aiming to reduce the risk of cataract formation (33). Both retinas received the prescribed dose of 20 Gy due to their close proximity to the target volume. All patients received concomitant high-dose intravenous corticosteroid therapy, administered according to the institutional protocol in use at the time of treatment.

Statistical analysis was performed for the data by R Core Team (2024) Language and Environment for Statistical Computing, Foundation for Statistical Computing, Vienna, Austria.

The study was approved by the Regional and Institutional Research Ethics Committee of the University of Debrecen (RKEB/IKEB 5585-2020).

## 3 Results

Of the 152 patients who received ORT between 2004 and 2020, 97 patients were available for follow up. For these 194 orbits, median follow-up time was 60 (IQR:38-100) months. Median CAS was 5 (IQR:4–6) before ORT and decreased to 2 (IQR:1–3) after ORT. Patient characteristics are shown in **Table 1**.

**Table 1:** Clinical characteristics of the patients.

Clinical parameter	Patient data
Female: male	78: 19 (n)
Age at the time of radiotherapy	53 (47-59) years (median, IQR)
Patients with diabetes (n)	19
Type 1 diabetes mellitus (n)	1
Type 2 diabetes mellitus (n)	18
Smokers (n)	29
Non-smokers (n)	35
Stopped smoking (n)	8
Unknown (n)	26
Follow up time (time between first radiotherapy and last ophthalmic examination)	60 (38-100) months (median, IQR)
Duration of thyroid disease at the time of irradiation	2 (1-8) years (median, IQR)
Time between the diagnosis of moderate to severe TED and radiotherapy	1 (1-3) years (median, IQR)
TSH before irradiation	0.84 (0-1.41) mU/L, (median IQR) (normal: 0.40-.4.20 mIU/L)
fT4 before irradiation	18 (12.15-25.40) pmol/L (median IQR) (normal: 10.30-24.50 pmol/L)
fT3 before irradiation	6.5 (4.4-11.0) pmol/L (median IQR) (normal:2.40-6.30 pmol/L)
Duration of diabetes (n=19) HbA1c before radiotherapy HbA1c after radiotherapy	16 (11.75-17.0) years (median IQR) 6.55 (6.22-6.95) mmol/L (median, IQR) 6.7 (6.30-7.15) mmol/L (median, IQR)

TED, thyroid eye disease; TSH, thyroid stimulating hormone; fT4, free thyroxine; fT3, free Triiodothyronine; HbA1c, hemoglobin A1C.

Of patients who received ORT, 19 had diabetes; 18 T2DM and one T1DM. Median duration of diabetes was 16 (IQR:11.75-17.0) years. Their HbA1c was in the target range, 6.55 (IQR:6.22-6.95) mmol/L and 6.7 (IQR:6.30-7.15) mmol/L before and after ORT, respectively.

As required by the inclusion criteria, no retinopathic lesions were observed before treatment. By the end of the follow-up period after ORT, no patient (0/97) developed retinopathy (median follow-up 60 month, IQR:38-100). Cataract was present in 12 (12.37%) patients before radiotherapy and in 31 (31.96%) after treatment. During the follow up period, no secondary orbital malignancies have developed. (**Table 2**)

**Table 2:** The effect of ORT and potential side effects of ORT.

Findings on follow up	Eyes/orbits involved
Retinopathy before radiotherapy Retinopathy after radiotherapy	right eye:0/97 left eye:0/97 right eye:0/97 left eye:0/97
Cataract/cataract surgery before radiotherapy Cataract/cataract surgery after radiotherapy	right eye:12/97 left eye:12/97 right eye:30/97 left eye:31/97
Eye/orbital regional malignancy	right 0/97 left 0/97
CAS before irradiation (n=196) CAS after irradiation (n=196)	5 (range: 4-6) 2 (range 1-3)

CAS, Clinical Activity Score.

## 4 Discussion

ORT is a non-specific anti-inflammatory treatment, which was first documented in 1936 (34). Probable mechanisms of action are suppression of orbital autoimmunity and limitation of connective tissue expansion (35). Intraorbital lymphocytes are particularly sensitive to radiation, and irradiation can also affect the innate immune system (36). Furthermore, radiotherapy reduces orbital fibroblast proliferation and GAG production (37). First line immunosuppressive treatment in moderate to severe TED is intravenous corticosteroids that can be combined with ORT (19, 38).

Based on experience gathered in oncology, development of radiation retinopathy depends on total dose, size of each radiation fraction, use of concurrent chemotherapy, and comorbidities such as hypertension, diabetes and vascular disease. A total dose of up to 50 Gy is frequently considered the threshold for the development of radiation-induced retinopathy; radiogenic retinopathy develops in more than 50% of patients exposed to 65 Gy with a healthy retina. Radiation doses below 35 Gy are believed to rarely lead to retinopathy (39). The most radiosensitive region of the retina is the posterior pole (26).

Coexistence of diabetes and TED often poses a major therapeutic challenge as corticosteroid administration is a contraindication in inadequately controlled diabetes (40). Presence of diabetic retinopathy is a contraindication to ORT, whereas diabetes without retinopathy requires case-by-case risk-benefit consideration (41). Older studies utilizing cobalt-60 as the irradiation source, suggested that diabetes mellitus is a contraindication to ORT as retinopathy after 20 Gy ORT occurred in diabetic patients (25). More recent studies, performed in the linear accelerator era, found post-ORT retinopathy neither in non-diabetic patients (42), nor in patients with diabetes (18). Our results are consonant with a study with median follow-up of 9.0 years, which detected no radiation-induced retinopathy in 121 patients, 10% of whom had diabetes (36).

In selected moderate-to-severe TED patients, especially in those whose principal feature is progressive diplopia, orbital irradiation is the preferred treatment (9, 10). However, the potential harm by induction of retinopathy limits its use, or at least requires profound consideration of the benefit-potential harm ratio. In the present study, 97 patients received 20 Gy irradiation by a linear accelerator, in combination with iv. corticosteroids for moderate to severe, active TED. No patient had developed retinopathy. The lack of retinopathy in the diabetes group may be related to the 3D irradiation planning or the well-managed diabetes based on HbA1c values, or the combination of both.

There are three differences compared to the previous studies. In the present study (i) all patients received iv. glucocorticoids during the ORT, (ii) a linear accelerator was used in all cases, and (iii) 3D computer-aided design was applied during pre-simulation. The latter enabled us to reduce the radiation dose received by the retina. Our results suggest that ORT, performed by a linear accelerator with 3D planning, and in combination with corticosteroids, is a safe treatment option for active TED in patients with or without diabetes.

One limitation of our study is its retrospective nature. Further, the diabetic patients were all of low risk for retinopathy due to excellent HbA1c values and the absence of uncontrolled hypertension. Uncontrolled diabetes and hypertension were considered relative contraindications for ORT. The low number of TED patients with diabetes may have been insufficient to detect a low-probability event. Finally, although the follow up period may have been sufficient to detect retinopathy, cataract formation and radiation induced tumor development may occur over a longer time.

In conclusion, the risk of radiation-induced retinopathy after orbital irradiation for TED is low. Diabetes with good glycemic control and without existing diabetic retinopathy may not be a contraindication if linear accelerator with 3D planning is used. Ophthalmic surveillance, particularly dilated fundus examination, is still recommended.

## Data Availability

The raw data supporting the conclusions of this article will be made available by the authors, without undue reservation.

## References

[1] Bartalena L Piantanida E Gallo D Lai A Tanda ML . Epidemiology, natural history, risk factors, and prevention of graves’ Orbitopathy. Front Endocrinol (Lausanne). (2020) 11:615993. doi: 10.3389/fendo.2020.615993, PMID: 33329408 PMC7734282

[2] Bahn RS . Graves’ Ophthalmopathy. N Engl J Med. (2010) 362:726–38. doi: 10.1056/NEJMra0905750, PMID: 20181974 PMC3902010

[3] Chng C Zheng K Kwee AK Lee MH Ting D Wong CP . Application of artificial intelligence in the assessment of thyroid eye disease (TED) - a scoping review. Front Endocrinol. (2023) 14:1300196. doi: 10.3389/fendo.2023.1300196, PMID: 38174334 PMC10761414

[4] Chin YH Ng CH Lee MH Koh JWH Kiew J Yang SP . Prevalence of thyroid eye disease in Graves ‘ disease : A meta-analysis and systematic review. Clin Endocrinol (Oxf). (2020) 93:363–74. doi: 10.1111/cen.14296, PMID: 32691849

[5] Scarabosio A Surico PL Singh RB Tereshenko V Musa M Esposito FD . Thyroid eye disease : advancements in orbital and ocular pathology management. J Pers Med. (2024) 14:776. doi: 10.3390/jpm14070776, PMID: 39064030 PMC11278049

[6] Verity DH Rose GE . Acute thyroid eye disease (TED): Principles of medical and surgical management. Eye. (2013) 27:308–19. doi: 10.1038/eye.2012.284, PMID: 23412559 PMC3597885

[7] Pelewicz-Sowa M Miśkiewicz P . Dysthyroid optic neuropathy: emerging treatment strategies. J Endocrinol Invest. (2023) 46:1305–16. doi: 10.1007/s40618-023-02036-0, PMID: 36802028 PMC10261203

[8] Ludgate M . Fibrosis in dysthyroid eye disease. Eye. (2019) 34:279–84. doi: 10.1038/s41433-019-0731-5, PMID: 31844169 PMC7002441

[9] Bartalena L Kahaly GJ Baldeschi L Dayan CM Eckstein A Marcocci C . The 2021 European Group on Graves’ orbitopathy (EUGOGO) clinical practice guidelines for the medical management of Graves’ orbitopathy. Eur J Endocrinol. (2021) 185:G43–67. doi: 10.1530/EJE-21-0479, PMID: 34297684

[10] Burch HB Perros P Bednarczuk T Cooper DS Dolman PJ Leung AM . Management of thyroid eye disease: a consensus statement by the American Thyroid Association and the European Thyroid Association. Eur Thyroid J. (2022) 41:1111–4. doi: 10.3760/cma.j.cn115989-20230512-00175 PMC9727317 36479875

[11] Taylor PN Zhang L Lee RWJ Muller I Ezra DG Dayan CM . New insights into the pathogenesis and nonsurgical management of Graves orbitopathy. Nat Rev Endocrinol. (2020) 16:104–16. doi: 10.1038/s41574-019-0305-4, PMID: 31889140

[12] Beige A Boustani J Bouillet B Truc G . Management of Graves’ ophthalmopathy by radiotherapy: A literature review. Cancer Radiother. (2024) 28:282–9. doi: 10.1016/j.canrad.2023.09.004, PMID: 38906800

[13] Drui D Du Pasquier Fediaevski L Vignal Clermont C Daumerie C . Graves’ orbitopathy: Diagnosis and treatment. Ann Endocrinol (Paris). (2018) 79:656–64. doi: 10.1016/j.ando.2018.08.005, PMID: 30177259

[14] Kim JW Han SH Son BJ Rim TH . Efficacy of combined orbital radiation and systemic steroids in the management of Graves ‘ orbitopathy. Greafes Arch Clin Exp Ophthalmol. (2016) 254:991–8. doi: 10.1007/s00417-016-3280-7, PMID: 26876240

[15] Oeverhaus M Witteler T Lax H Esser J Führer D Eckstein A . Combination therapy of intravenous steroids and orbital irradiation is more effective than intravenous steroids alone in patients with graves ‘ Orbitopathy. Horm Metab Res. (2017) 49:739–47. doi: 10.1055/s-0043-116945, PMID: 28922676

[16] Marcocci C Bartalena L Bogazzi F Lepri A Pinchera A . Orbital radiotherapy combined with high dose systemic glucocorticoids for Graves ‘ ophthalmopathy is more effective than radiotherapy alone : results of a prospective randomized study. J Endocrinol Invest. (1991) 14:853–60. doi: 10.1007/BF03347943, PMID: 1802923

[17] Li YJ Luo Y He WM Li P Wang F . Clinical outcomes of graves’ ophthalmopathy treated with intensity modulated radiation therapy. Radiat Oncol. (2017) 12:1–8. doi: 10.1186/s13014-017-0908-7, PMID: 29110673 PMC5674803

[18] Marcocci C Bartalena L Rocchi R Marinò M Menconi F Morabito E . Long-term safety of orbital radiotherapy for Graves’ ophthalmopathy. J Clin Endocrinol Metab. (2003) 88:3561–6. doi: 10.1210/jc.2003-030260, PMID: 12915636

[19] Adams EJ Warrington AP . A comparison between cobalt and linear accelerator-based treatment plans for conformal and intensity-modulated radiotherapy. Br J Radiol. (2008) 81:304–10. doi: 10.1259/bjr/77023750, PMID: 18250119

[20] Bartalena L Marcocci C Manetti L Tanda ML Dell’Unto E Rocchi R . A. Orbital radiotherapy for graves’ ophthalmopathy. Thyroid. (2002) 12:245–50. doi: 10.1089/105072502753600223, PMID: 11952048

[21] Healy BJ van der Merwe D Christaki KE Meghzifene A . Cobalt-60 machines and medical linear accelerators : competing technologies for external beam radiotherapy. Clin Oncol. (2016) 29:802–9. doi: 10.1016/j.clon.2016.11.002, PMID: 27908503

[22] Uzun S Toyran S Akay F Gundogan FC . Delayed visual loss due to radiation retinopathy. Pakistan J Med Sci. (2016) 32:516–8. doi: 10.12669/pjms.322.9221, PMID: 27182273 PMC4859056

[23] Sobel RK Aakalu VK Vagefi MR Foster JA Tao JP Freitag SK . Orbital radiation for thyroid eye disease: A report by the American academy of ophthalmology. Ophthalmology. (2021) 129:450–5. doi: 10.1016/j.ophtha.2021.10.025, PMID: 34895729

[24] Weissmann T Lettmaier S Donaubauer AJ Bert C Schmidt M Kruse F . Low- vs. high-dose radiotherapy in Graves’ ophthalmopathy: a retrospective comparison of long-term results. Strahlentherapie und Onkol. (2021) 197:885–94. doi: 10.1007/s00066-021-01770-9, PMID: 33860819 PMC8458186

[25] Wakelkamp IMMJ Tan H Saeed P Schlingemann RO Verbraak FD Blank LECM . Orbital irradiation for Graves’ ophthalmopathy: Is it safe? A long-term follow-up study. Ophthalmology. (2004) 111:1557–62. doi: 10.1016/j.ophtha.2003.12.054, PMID: 15288988

[26] Seregard S Pelayes DE Singh AD . Radiation therapy: Posterior segment complications. Dev Ophthalmol. (2013) 52:114–23. doi: 10.1159/000351088, PMID: 23989132

[27] Yang J Liu Z . Mechanistic pathogenesis of endothelial dysfunction in diabetic nephropathy and retinopathy. Front Endocrinol (Lausanne). (2022) 13:816400. doi: 10.3389/fendo.2022.816400, PMID: 35692405 PMC9174994

[28] Godfrey KJ Kazim M . Radiotherapy for active thyroid eye disease. Ophthal Plast Reconstr Surg. (2018) 34:S98–S104. doi: 10.1097/IOP.0000000000001074, PMID: 29771752

[29] Gardiner TA Archer DB Silvestri G Amoaku WM . Radiation and diabetic retinopathy : A dark synergy. Int J Transl Med. (2023) 3:120–59. doi: 10.3390/ijtm3010011

[30] Mourits MP Prummel MF Wiersinga WM Koornneef L . Clinical activity score as a guide in the management of patients with Graves’ ophthalmopathy. Clin Endocrinol (Oxf). (1997) 47:9–14. doi: 10.1046/j.1365-2265.1997.2331047.x, PMID: 9302365

[31] Bentzen S Constine LS Deasy JO Eisbruch A Jackson A Marks LB . Quantitative analyses of normal tissue effects in the clinic (QUANTEC): an introduction to the scientific issues. Int J Radiat Oncol Biol Phys. (2010) 76:3–9. doi: 10.1016/j.ijrobp.2009.09.040, PMID: 20171515 PMC3431964

[32] Lawrence YR Li XA Naqa I Hahn CA Marks LB Merchant TE . Radiation dose-volume effects in the brain. Int J Radiat Oncol Biol Phys. (2010) 76:20–7. doi: 10.1016/j.ijrobp.2009.02.091, PMID: 20171513 PMC3554255

[33] Nguyen SM Sison J Jones M Berry JL Kim JW Murphree AL . Lens dose-Response prediction modeling and cataract incidence in patients with retinoblastoma after lens-Sparing or whole-Eye radiation therapy. Int J Radiat Oncol Biol Phys. (2019) 103:1143–50. doi: 10.1016/j.ijrobp.2018.12.004, PMID: 30537543 PMC6667833

[34] Igersheimer J . Visual changes in progressive exophthalmos. Am J Ophthalmol. (1955) 53:94–104. doi: 10.1001/archopht.1955.00930010096010, PMID: 13217551

[35] San I Arenas M Carmona R Rutllan J Medina-rivero F Lara P . Review of the treatment of Graves ‘ ophthalmopathy. role New Radiat techniques. Saudi J Ophthalmol. (2018) 32:139–45. doi: 10.1016/j.sjopt.2017.09.003, PMID: 29942184 PMC6010590

[36] Tanda ML Bartalena L . Efficacy and safety of orbital radiotherapy for graves’ orbitopathy. J Clin Endocrinol Metab. (2012) 97:3857–65. doi: 10.1210/jc.2012-2758, PMID: 22962421

[37] Verrienti M Gagliardi I Valente L Stefanelli A Borgatti L Franco E . Late orbital radiotherapy combined with intravenous methylprednisolone in the management of long-lasting active graves ‘ orbitopathy : a case report and literature review. Endocrine. (2024) 85:576–83. doi: 10.1007/s12020-024-03788-2, PMID: 38517640 PMC11291534

[38] Bartalena L Baldeschi L Dickinson A Eckstein A Kendall-Taylor P Marcocci C . Consensus statement of the European Group on Graves’ orbitopathy (EUGOGO) on management of GO. Eur J Endocrinol. (2008) 158:273–85. doi: 10.1530/EJE-07-0666, PMID: 18299459

[39] Reichstein D . Current treatments and preventive strategies for radiation retinopathy. Curr Opin Ophthalmol. (2015) 26:157–66. doi: 10.1097/ICU.0000000000000141, PMID: 25730680

[40] Urselli F Pontieri G Peschi L Liccardi A Ruggiero AR Biondi B . Active moderate-to-severe graves’ orbitopathy in a patient with type 2 diabetes mellitus and vascular complications. Front Endocrinol (Lausanne). (2019) 9:810. doi: 10.3389/fendo.2018.00810, PMID: 30705666 PMC6344418

[41] Marquez SD Lum BL McDougall IR Katkuri S Levin PS MacManus M . Long-term results of irradiation for patients with progressive Graves’ ophthalmopathy. Int J Radiat Oncol Biol Phys. (2001) 51:766–74. doi: 10.1016/S0360-3016(01)01699-6, PMID: 11697323

[42] Nicosia L Reverberi C Agolli L Marinelli L De Sanctis V Minniti G . Orbital radiotherapy plus concomitant steroids in moderate-to-severe graves’ ophthalmopathy: Good results after long-term follow-up. Int J Endocrinol Metab. (2019) 17:1–7. doi: 10.5812/ijem.84427, PMID: 30881470 PMC6408739

